# Mapping ‘Occupational Health’ courses in India: A systematic review

**DOI:** 10.4103/0019-5278.58917

**Published:** 2009-12

**Authors:** S. P. Zodpey, Himanshu Negandhi, R. R. Tiwari

**Affiliations:** Public Health Education, Public Health Foundation of India, New Delhi, India; 1 National Institute of Occupational Health, Ahmedabad, India

**Keywords:** Human resource, occupational health, systematic review, training capacity

## Abstract

The occupational health scenario is undergoing a paradigm shift in developing countries with rapid industrialization. Inadequate human resource is, however, a concern. The creation of Basic Occupational Health Services will demand a further increase in specialist manpower. The current training capacity of occupational health specialists has been mapped by a systematic review in India. Twenty-one institutes have been identified all across the country. They have an existing capacity for training about 460 specialists. This number is inadequate considering the population of India's working class. A mixture of strategies must be urgently planned for addressing this issue.

## INTRODUCTION

Working adults represent half the world's population. As the work environment constitutes over a third of the time spent in their lives by these adults during their lifespan and significantly contributes to their overall individual health, it is important to ensure a healthy working atmosphere. Productivity at work is directly influenced by the health status of the workers. An unhealthy workforce is an impediment to increasing workplace productivity, thus affecting the overall national productivity. Poor occupational health and reduced working capacity of the workers may cause an economic loss of up to 10-20% of the Gross National Product (GNP). The cost to society has been estimated at 2-14% of the GNP in different studies, in different countries.[3] The WHO estimates that only 10-15% of the workers have access to basic occupational health services. The burden of disease attributed to occupational diseases is high and it is estimated to be about 11 million cases annually, with about 700,000 deaths.[4] According to a World Bank estimate, two thirds of the occupationally determined loss of disability-adjusted life years (DALYs) could be prevented by occupational health and safety programs.[5] A healthy workforce is beneficial to both, the industry and the country.

The occupational health scenario is undergoing a paradigm shift with rapid industrialization in the developing countries. Many developing countries in Asia, including India, have had an expansion in international trade over the past decade. The Indian trade boom is attributed to economic liberalization. With over 40 million belonging to the working population,[6] India has a very large population base engaged in industrial activity. This group is at a special risk of occupational hazards and diseases. The health needs of these populations also differ according to the industry of work. The knowledge and orientation for diagnosing such occupation-specific conditions are evolving globally in the form of a specialty health service.

This evolution is facilitated by several factors. The human resource engaged in occupational health services is one important factor. However, it is deficient in most developing countries.[7] Regular courses at graduation or post-graduation levels for personnel such as occupational hygienists, safety officers, occupational health nurses, ergonomists, and so on, are rare and generally lacking in details.[7] The establishment of training services in occupational health could help to improve the manpower scenario in such situations. While there has been a slow but definite increase in the manpower for provision of general health care services to the population, the rate of increase of occupational health services has been slow till 2004. There has been renewed interest in the creation of Basic Occupational Health Services (BOHS), which is an application of primary health care to occupational health. This is further expected to fuel demand for trained manpower in this field.

There are around 1000 qualified occupational health professionals in India and only around 100 qualified hygienists.[8] The current estimated need for occupation health specialists in the country is much higher and there is a significant gap in the demand and supply of this specialist service. This need has been expressed at several conferences and forums. The gap has already prompted a renewed interest in a variety of specialist courses directed toward fresh medical graduates, industrial doctors, engineers, and regular work staff. This is expected to cater to the preventive and medical needs of the group. An analysis of the existing teaching and training services in occupational health would reveal this shortcoming and help plan for the future establishment of need-based responsive courses. This article is directed toward examining the current status of occupational health training in the country, with a focus on the variety of courses offered and the capacity for the production of trained man-power at the national level.

## MATERIALS AND METHODS

The data regarding the existing courses in occupational health and related fields in the country was obtained using three strategies. A systematic, pre-defined approach was used for obtaining this information. Each step was conducted in a parallel manner, and the information was entered into an excel spreadsheet. The first search strategy comprised of using the information available on the internet. The internet search was conducted using the Google search engine. The first step in this strategy involved identifying a set of key words encompassing various domains related to occupational health. The key words included were *ergonomics, industrial health, industrial hygiene, industrial medicine, industrial toxicology, occupational health, and occupational medicine*. The search was limited to courses offered in India and to collaborations between Indian and foreign institutes if any. The websites of the Ministry of Environment and Ministry of Labor were searched using the key words of the identified subjects and training programs. A similar search was conducted through the websites of the International Labor Organization (ILO), World Health Organization (WHO), International Commission on Occupational Health (ICOH), and the Indian Association of Occupational Health (IAOH). The website of the Directorate General, Factory Advice and Labor Institutes, India (DGFASLI) was also accessed for information on training programs. The search was not restricted by course duration or the type of degree/certification awarded on successful completion. Detailed information about the courses was collected from the respective institutions or from the designated websites of these institutions. Short-term courses offered by various institutions, lasting from a few days to a few weeks were disregarded and not entered into the matrix.

The second strategy involved a detailed literature review of the occupational health courses. Indexed and non-indexed journals in the field were identified and searched for notifications and invitation of nominations for educational courses. Key institutes involved in research in occupational health were identified from the author affiliations. Key cross-references were also identified from the articles and referred for relevant information.

The third strategy involved contacting experts in the field of occupational health in India. This was done through e-mail and/or telephone. They were requested to share information about the courses offered in their institutes and also to suggest the names of other institutes offering courses in the identified fields. A snowballing approach was used till no new institution or course name surfaced. If at the end of the three strategies any information regarding the courses was unavailable, it was left blank in the matrix.

The search was directed at obtaining data on the following parameters: Name and location of the institute offering the course, theme and course duration, course structure, eligibility criteria, and the capacity of training. These parameters were incorporated in a matrix. The institutional data was entered in this matrix and the findings were triangulated wherever possible using alternative strategies. Any other salient features of relevance to the courses were also incorporated subjectively into this matrix.

## RESULTS

The data collected was inserted in the matrix as described earlier in the methods. The course descriptions have been listed in Table 1.

**Table 1:** Occupational and Environmental health courses in India

Institute	City	Course	Duration	Theme	Type of course	No. of students	Eligibility criteria
Central Labor Institute, Mumbai	Mumbai	Associate Fellow of Industrial Health	3 months	Industrial health	Certificate course	50	MBBS Degree from an Institution recognized by the Medical Council of India. Minimum one year experience in Occupational health related work in an industry or 2 years in general practice after the date of completion of internship
Regional Labor Institute	Calcutta	Associate Fellow of Industrial Health	3 months	Industrial health	Certificate course	25	
State factory inspectorate	Goa	Associate Fellow of Industrial Health	3 months	Industrial health	Certificate course	25	
Model Center on Occupational Health, BHEL	Trichy	Associate Fellow of Industrial Health	3 months	Industrial health	Certificate course	25	
Lokmanya Medical Research center	Pune	Associate Fellow of Industrial Health	3 months	Industrial health	Certificate course	25	
Center for Occupational and Environmental Health, Maulana Azad Medical College	New Delhi	Associate Fellow of Industrial Health	3 months	Industrial health	Certificate course	25	
Regional Labor Institutes	Mumbai	Advanced Diploma Course	1 year fulltime	Industrial safety	Diploma	50	Recognized Degree or Diploma in Technology/Engineering or recognized Degree in Physics or Chemistry with practical experience in a supervisory capacity (variable for degree and diploma holders), or safety department in industry or
	Kolkata	Post Graduate Diploma in Industrial Safety	1 year fulltime	Industrial safety	Diploma	50	
	Chennai	Diploma in Industrial Safety	1 year fulltime	Industrial safety	Diploma	50	Research, training education in the field of Industrial Safety, or in a Government
	Kanpur	Post Graduate Diploma in Industrial Safety	1 year fulltime	Industrial safety	Diploma	50	Department in administration, or in construction industry
National Institute of Occupational Health (NIOH) 1966	Ahmedabad	PhD	3 years	Occupational health	Doctorate	-	MD
		Certificate in Industrial Health	3 months	Industrial health	Certificate	15-20	MBBS
Indian Institute of Toxicological Research (earlier ITRC)	Lucknow	Summer trainings	3-6 months	Toxicological research	-	-	Students of M.Sc
All India Institute of Hygiene and Public Health	Calcutta	Diploma in Industrial Health	2 years	Industrial health	Diploma	2-3	MBBS
Sri Ramachandra Medical College and Research Institute	Chennai	M.Sc. (distance education)	3 years	Industrial hygiene and safety	Post graduate	20	B.E./B.Tech. (Mechanical, Electrical, Sanitary, Chemical, Civil, Mining or Industrial Engineering)/M.B.B.S./B. Sc. (Physics, Chemistry, Zoology, Environmental Sciences or Allied Health Sciences or B.Sc. Nursing or any recognized Degree related to Health/ Environmental Sciences
		PG Certificate course in Industrial Health	1 year	Industrial health	Certificate	20	M.B.B.S. Degree and permanent registration with any of the State Medical Council of India
Institute of Science, Training and Advanced Research (previously BVM Engineering College)	Anand	Masters in Industrial Hygiene	2 years full time	Industrial hygiene and safety	Masters	15	Any Science Graduate
British Safety Council	London	International Diploma	One month	Occupational safety and health	Diploma	-	Either a Certificate in Occupational Safety and Health or an alternative qualification in a related sector or appropriate experience in a health and safety role. Literacy and numeracy skills adequate to cope with the course. IELTS may be necessary
Amrita Institute of Medical Sciences	Kochi	PhD	3 years	Occupational health	Doctorate	-	MD
National Institute of Industrial Engineering (NITIE)	Mumbai	PG program in Industrial safety and environmental management (PGDISEM)	24 months	Industrial safety and environmental management	-	-	-
		Diploma in Ergonomics (DErg)	9 months	Ergonomics	Diploma	-	Ergonomics practitioners, industrial engineers, safety professionals, occupational therapists, physical therapists, orthopedic surgeons and human resource personnel
Armed Forces Medical College, Pune	Pune	Diploma in Industrial Health	2 years	Industrial health	Diploma	01	MBBS; only for in-service candidates with the armed forces
Annamalai University	Chennai	Diploma in Industrial Hygiene	2 years (Distance education)	Industrial hygiene	Diploma	-	Any Science graduate
Mahatma Gandhi Labor Institute	Ahmedabad	Diploma in Industrial Safety	2 years Full time	Industrial safety	Diploma	15-20	Any Science Graduate
Pramukhswami Medical college	Karamsad	Diploma in Industrial Hygiene	2 years Full time	Industrial hygiene	Diploma	01	M.B.B.S.

There are twenty-one institutes in India that offer courses in the identified fields. With the exception of the All India Institute of Hygiene and Public Health-Calcutta, all other institutes have been established after 1950. The institutes are widely distributed, with the maximum representation by the western region of the country. The entry into a majority of these institutes is open on a national level. However, each institute has its own unique entry level qualification and eligibility criteria. The admission procedure for each institute is independent of the other institutes. Government as well as private institutes are involved in the teaching and training of occupational health-related disciplines.

The courses offered vary from short-term trainings, certificate courses, diploma courses, master's level courses, post-graduate diploma courses, and doctorate degrees. The courses are conducted in a distance education format (correspondence programs) as well as full-time residential courses. The course durations range from a few weeks for short-term training to a full year for a diploma course. The M.Sc. (Masters of Science) course lasts for three years. The PhD (doctorate) programs conducted by the institutes can be completed in a minimum of three years. The maximum courses offered in this field, irrespective of the degree granted, last for two years. Very few courses extend beyond this duration.

Courses are offered in a wide variety of sub-specialties, which include, industrial health, occupational health, occupational and environmental medicine, occupational safety and health, ergonomics, industrial safety, and environmental management. The most frequently covered sub-specialties are industrial health and occupational health. The other specialties are offered only in select institutions.

The eligibility criteria for the courses vary as per the job responsibilities in the industry. Recognized degree or diploma holders in a Technology/Engineering branch or in physics/chemistry with a practical experience in a supervisory capacity are eligible for the one-year, full-time Advanced Diploma course in Industrial Health offered by the Regional Labor Institutes. With a lower academic qualification such as a Diploma, the duration of the pre-requisite work experience is increased from two to five years. In addition to the currently employed working staff, researchers and teachers are also eligible for the Advanced Diploma course. Medical graduates are eligible for a Certificate course in Industrial health, conducted by the Regional Labor Institutes. The basic requirement is the possession of a M.B.B.S. degree, recognized by the Medical Council. They must also have at least one year of experience in occupational health or at least two years of experience in general practice.

A nine-month long Diploma in Ergonomics is offered at the National Institute of Industrial Engineering (NITIE), Mumbai. The target audience for this course includes ergonomics practitioners, industrial engineers, safety professionals, occupational therapists, physical therapists, orthopedic surgeons, and human resource personnel. The British Safety Council offers the opportunity for advanced training in occupational health by offering an international diploma in occupational safety and health. The course needs a prior academic qualification in Occupational Safety and Health or experience in a health and safety role. Proficiency in the English language may be demanded if the medium of instruction is non-English. Their course is said to be in accordance with the requirements of the Institute of Occupational Safety and Health (IOSH) and the Institute of Environmental Management and Assessment (IEMA).

A two-year Diploma in Industrial Hygiene is offered through distance education by the Annamalai University. This course is open for all science graduates. A Masters in Industrial Safety and Hygiene is offered to any science graduate by the BVM college of Engineering. This is a full-time course, which lasts for two years.

The potential yearly output in the country varies for the various types of courses. The maximum human resource that can be generated annually is 50 from the Central Labor Institute and the Regional Labor Institutes.

## DISCUSSION

Preventive services are globally encouraged by all decision makers. However, there is a wide variation in the extent of services provided. Many countries have adopted a basic primary health service approach through the government machinery as signatories to the Alma Ata conference.[9] These services are provided at affordable rates and tailored for wide acceptance. India has a similar health care system, run by the local governments from taxpayer costs. The Indian foray into universal health care was first propounded by the Bhore committee report in 1946[10] and reinforced by the Alma Ata Declaration. Occupational health services are an essential component of this general health care, but have not yet been completely incorporated into the public health system. The provision of these services to the working population is also a dimension of primary health care.

The hazards to which the working population is exposed at the workplace necessitates special attention to occupational health issues; preventive as well as curative. This necessity stems from the specialized screening, diagnosis, and provision of care for occupational diseases, the planning and designing of characteristics for ergonomically safe workplaces, and the development of preventive strategies for specific occupations. While the state is responsible for the overall health status of its population, the responsibility for the health of the workforce is shared by the state and the employer in varied proportions across the world, including India. The provision of these occupational health services, however, shows a high degree of variation.

In India, this responsibility of provision of occupational health services is governed by specific legislations.[11] These include The Factories Act, 1948, The Mines Act, 1952, and The Dock Workers (Safety, Health, and Welfare) Act, 1986. Other legislations such as the Workmen's Compensation Act, 1923, and the Employees State Insurance Act, 1948, are directed toward compensation after injury, disease or accident. However, for the effective service provision, the legislations must be adequately complemented by the creation of an appropriate infrastructure and enabling an environment for policy implementation. With an estimated 17 million occupational, non-fatal injuries (17% of the world) and 45,000 fatal injuries[12] (45% of the total deaths due to occupational injuries in the world), India significantly contributes to the global occupational injury scene. India is also estimated to account for 1.83 million cases of occupational diseases.[13] However, out of an estimated 500 million workers in India, only about 5-10% of them have access to occupational health services.[14]

The provision of optimum occupational health services is estimated to require one physician and two nurses per 5000 workers; with a wide variation, depending on the branch of industry and workplaces, as well as, the geographical distribution.[15] The estimated 500 million workers would need a staggering 100,000 doctors! The number of allopathic doctors possessing recognized medical qualifications (under the IMC act) and registered with state medical councils for the year 2005 was only 660,801.[16] This highlights the fact that although the creation of a new specially trained cadre can continue, there must be a strong motivation for the inclusion of occupational health training in the very ethos of general health care. Although, no definitive data on the exact number of doctors working in occupational health services on a full time/part time status or the level of qualification/training, is available for India, this number is expected to be low. The ratio of occupational health physicians to 100,000 workers is 61, 26, and five in Finland, Netherlands, and UK, respectively.[17] For provision of these services widely, the above-mentioned national statistics warrant a renewed interest in the creation of human resource.

The potential training capacity per annum in India for occupational health is about 460 specialists. Experience has documented that even this capacity is hardly achieved. In contrast, nearly 400 candidates appear for examinations in all courses related to occupational health in the United Kingdom.[18] This in-spite of the fact that India's population is more than 18 times that of the United Kingdom.[19] The number of doctors and occupational health practitioners is even higher than the UK numbers in other European countries. This consequently highlights that it will take an estimated 55 additional years for India to achieve the existing UK standards of five occupational health specialists for 100,000 workers at the current training capacity, assuming zero population growth! Addressing this shortfall has obvious challenges in developing countries like India. Budgetary allocations to the health sector not-withstanding, there are other issues in health care that also warrant immediate attention and resource allocation. Occupational health is not as firmly advocated or recognized as infant and child care or nutrition. However, as a sub-group, it seeks to serve a very large estimate of 500 million of the population. By its sheer size, it forces us, as public health activists, to renew the focus on this sector. Two thrust areas can be identified for future action. The first would be to inculcate and strengthen the essentials of occupational health services in the current manpower. The second strategy could be directed toward the creation of newer facilities that translate into an increased number of specialists.

The strategies can be rolled out in three time-frames. The first phase of this strategy would be to review and tailor the existing curriculum of M.B.B.S. in the country. They form the current mainstay in the field of occupational health. A majority of the doctors in the industry possess M.B.B.S and receive a certification after a brief course, as mandated by the law. Nevertheless, a wider dissemination of the principles of occupational health into the routine curriculum could help create a strong background for this field in the doctors. Currently the concepts related to occupational health are covered in the medical syllabus by the Departments of Preventive and Social Medicine during undergraduate as well as postgraduate studies. The impetus on the field varies between medical colleges, with several students even pursuing their postgraduate thesis in this field. No concrete estimates of the number of hours that must be devoted to teaching in this field are available. A formalization of the same can be sought as a part of the essentials in the curriculum. A structured curriculum that is responsive to modern day issues at the workplace would find great support. Even the existing teaching in the field can be of an integrated approach. It can involve all specialties such as physiotherapy, surgery, orthopedics, and general medicine to teach about occupational injuries and their management. This holistic approach to the teaching would impart a comprehensive approach to patient care rather than the fragmented and compartmentalized pattern of conventional teaching. Another step in the short term could be the initiation of new certification courses, by expanding the existing training capacity of the institutes. It is not easy for in-service practitioners to participate in long programs. The design of correspondence and web-blended programs are potential solutions to this vexing issue.

A medium term strategy could be toward de-medicalizing and increasing the participation of various engineering and industrial sectors toward this field. The current national scenario does involve options for safety officers involved in the industry. However, this role can be further strengthened and diversified to include additional decision-making responsibilities. This would ensure a wider collective participation that can be expected to contribute to the development of this field.

The creation of a resource consuming newer infrastructure will have to be contemplated in the long run. Instead of slating it for the distant future, a concerted approach could be made toward the initiation of newer courses. Sufficient experience exists in the national institutes with regard to the uptake and utilization of the seats in various programs. The industry is also clear about its requirements from the specialist practitioners. An open dialog between them, at the highest decision-making levels, can be initiated. It could proceed on a mandate of identifying the thrust areas and addressing the gaps. The Indian corporate sector could be identified as a significant partner in this endeavor and could possibly play a funding role in the development of this sector in the country. Ensuring healthcare for the workers will need a concerted effort by the various stakeholders. The present study seeks to highlight the human resource gap in occupational health and convincingly argues for an improvement in the current training capacity.
